# In Silico Screening of Natural Products Isolated from Mexican Herbal Medicines against COVID-19

**DOI:** 10.3390/biom11020216

**Published:** 2021-02-04

**Authors:** Nadia A. Rivero-Segura, Juan C. Gomez-Verjan

**Affiliations:** Dirección de Investigación, Instituto Nacional de Geriatría, Ciudad de Mexico 10200, Mexico; nrivero@inger.gob.mx

**Keywords:** COVID-19, SARS-CoV-2 virus, cichoriin, natural products, chemoinformatics, antiviral compounds, coumarin

## Abstract

The COVID-19 pandemic has already taken the lives of more than 2 million people worldwide, causing several political and socio-economic disturbances in our daily life. At the time of publication, there are non-effective pharmacological treatments, and vaccine distribution represents an important challenge for all countries. In this sense, research for novel molecules becomes essential to develop treatments against the SARS-CoV-2 virus. In this context, Mexican natural products have proven to be quite useful for drug development; therefore, in the present study, we perform an in silico screening of 100 compounds isolated from the most commonly used Mexican plants, against the SARS-CoV-2 virus. As results, we identify ten compounds that meet leadlikeness criteria (emodin anthrone, kaempferol, quercetin, aesculin, cichoriin, luteolin, matricin, riolozatrione, monocaffeoyl tartaric acid, aucubin). According to the docking analysis, only three compounds target the key proteins of SARS-CoV-2 (quercetin, riolozatrione and cichoriin), but only one appears to be safe (cichoriin). ADME (absorption, distribution, metabolism and excretion) properties and the physiologically based pharmacokinetic (PBPK) model show that cichoriin reaches higher lung levels (100 mg/Kg, IV); therefore, it may be considered in developing therapeutic tools.

## 1. Introduction

COVID-19 caused by the novel etiological agent SARS-CoV-2 has been diagnosed in more than 100 million people worldwide, causing the death of more than 2 million (until January 2021), and leading to a socio-economic crisis worldwide. At the time of publication, there are non-effective pharmacological treatments, and the logistics for the vaccine distribution still representing a challenge for developing countries, due to its conditions for its transportation and storage. In this context, besides the current vaccine development (AZD1222, developed by University of Oxford and AstraZeneca (Cambridge, U.K.); BNT162, developed by Pfizer (New York, NY, USA)/BioNTech (Mainz, Germany), and mRNA-1273, developed by Moderna Inc. (Cambridge, MA, USA), among others), other biotechnological strategies such as human monoclonal antibodies (47D11, B38 and H4) [1] or recombinant proteins (rbACE2 developed by Kafrelsheikh University, and RhACE2 APN01 by Apeiron Biologics(Vienna, Austria); Rhu-pGSN developed by BioAegis Therapeutics Inc. (San Diego, CA, USA)) that target key molecules involved in SARS-CoV-2 viral entry [2] have been proposed to be promising tools. However, they are still under research, and further analyses are needed. So, according to Twomey et al. [3], there are two primary strategies to counteract COVID-19. On one hand, there are the already mentioned biotechnological products, and on the other hand, repurposing existing drugs may be the most reliable tool to counteract against COVID-19 since these are already approved drugs or highly studied compounds that may be useful. In this sense, such a strategy becomes crucial since repurposing drugs is more straightforward and saves time and money unlike the development of novel drugs. Moreover, repurposing compounds is more accurate and leads to fewer failures in clinical phases than biotechnological products. Thus, most of them are already proved, leading to the fast development of novel therapeutic tools against COVID-19.

On the other hand, natural products from plants, animals and microorganisms represent an essential chemical source. According to Cragg and Newman [4,5,6], it is estimated that from 1981 to 2019 about 36.3% of small molecules clinically used (or recently approved) as antimicrobial, antiparasitic and anticancer treatment are based on natural products directly or as derivatives (semi-synthetic or prototypes for leader molecules), most of which are mainly isolated from plants (~20%) [7]. Interestingly, a novel FDA classification called “botanical drugs” consists of vegetable materials with complex mixtures that must pass quality chemical control and manufacturing validation process [6].

On the other hand, several efforts have been performed to identify whether natural products possess antiviral effects. For instance, aqueous extracts of *Ocimum basilicum* have proven to be effective against enterovirus by inhibiting viral replication [8]. Furthermore, saikosaponins [9], phenolic compounds, amentoflavone, myricetin and scutellarein isolated from *Lycoris radiata*, *Artemisia annya*, *Torreya nucifera* and *Lindera aggregata* are active against SARS-CoV-1 [10,11,12,13]. For a complete review of herbal medicine, please refer to [14]. Moreover, other sources of antiviral compounds have shown to possess such properties; for instance, Suwannarach et al. [15] suggest that fungi are a source of natural bioactive compounds that are potentially useful for preventing viral infections and improving human immunomodulation; additionally, natural products from marine species have recently shown important antiviral properties [16].

In this context, Mexico is the fourth country with the greatest biodiversity globally. It has been estimated that there are more than 30,000 species of plants; and after China, Mexico is the country with the second highest number of medicinal plants (4500 spp., approximately). Moreover, Mexican ethnomedicine has a deeply rooted tradition to use herbal remedies to treat the most common health problems. In this sense, Mexican plants have been studied phytochemically, pharmacologically and anthropologically for more than 100 years, representing a significant research line in Mexico and worldwide [17]. Interestingly, the most frequently used plants accordingly to [17,18,19] are *Opuntia ficus*, *Scoparia dulcis*, *Citrus aurantium*, *Prunus persica*, *Rosmarinus officinalis*, *Prunus persica*, *Rosmarinus officinalis*, *Equisetum hyemale*, *Tilia mexicana*, *Mentha piperita*, *Larrea divaricata*, *Taraxacum officinale*, *Morus alba*, *Verbascum densiflorum*, *Matricaria recutita*, *Urtica dioica*, *Passiflora incarmata*, *Tilia europea* and *Aloa Vera* most of which have shown several pharmacological properties such as antiparasitic, pain and menstrual pain relief, issues of the nervous system, among others.

On the other hand, computational tools and novel algorithms have been implemented over the last years to accelerate and optimize the drug discovery process (estimated in 20 years and about 1.3 billion USD [20]). Several methods have been shown to reduce drug development costs up to 50%, such as chemoinformatics, quantitative-structure activity relation (QSAR), docking, molecular similarity, network pharmacology and pharmacogenomics computational de novo design, to mention a few examples [21]. Moreover, the bioinformatic era has shown a significant increase in the development of computational and web tools that could be applied for novel drug design. For instance, systems pharmacology has enabled us to understand the dose-response relationships of novel compounds and perform physiologically based pharmacokinetic (PBPK) models that make it easy to understand the pharmacokinetics properties of novel compounds. Additionally, chemoinformatic approaches allow us to analyze databases of compounds to obtain information about its potential as drugs.

Hence, in an attempt to quickly propose the discovery of natural products that could be used against SARS-CoV-2, in the present study we performed a chemoinformatic analysis with 100 compounds isolated from the previously mentioned medicinal plants and some marine natural products reported in Mexico [22,23].

## 2. Materials and Methods

### 2.1. Chemical Descriptors and Computational Screening

**Figure 1:**
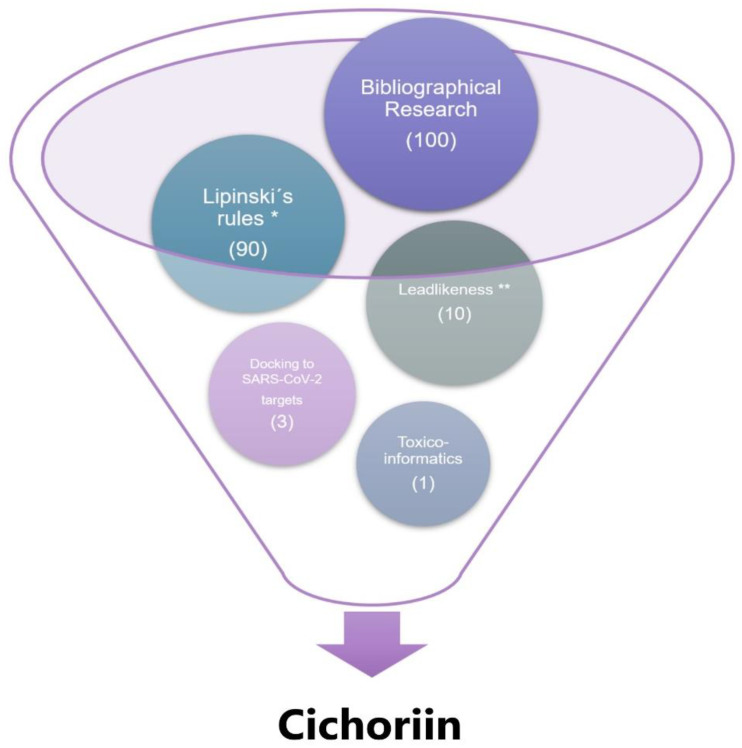
Funnel chart representing the steps followed toward compound selection. First, we performed bibliographical research that identified 100 potential compounds frequently used to treat the most common health problems. Then we tested such compound in silico, and only 90 compounds met Lipinski’s rules (* corresponding to one or zero Lipinski’s rules violations), and from these only ten compounds meet leadlikeness criteria (** corresponding to 250 ≤ MW ≤ 350, logP ≤ 3.5, # R-bounds ≤ 7 [27]). Lastly, docking approaches identify that only three compounds dock appropriately to the essential proteins of SARS-CoV-2 virus (RdRp (RTP site), Nsp14 (ExoN), Nsp3 (207-379, AMP site) and papain-like protease); however, only 1 (cichoriin) appears to be the safest for the human.

We obtained a list of the 100 natural products isolated from the most traditionally used plants, as stated by [18] and [17]. Additionally, we added ten essential marine natural products and already reported compounds with antiviral activities [22,23,24]. From such compounds, we calculate the chemoinformatic properties using the Osiris DataWarrior (freeware software, DataWarrior V4.7.2, Idorsia Pharmaceuticals Ltd., Allschwil, Switzerland), which calculates lipophilicity (expressed as compound logP), solubility in water (expressed as logS), molecular weight, druglikeness, leadlikeness and toxicoinformatic properties of compounds. Additionally, we used the Swiss Bioinformatics Institute, which possesses a web server that calculates several ADME (absorption, distribution, metabolism and excretion) properties that could help to delve into compounds’ pharmaceutical properties. The complete description of the computational medicinal chemistry algorithm for both was published [25]. We use consensus Log P from 5 different predictions; LogS (Silicos-IT) is a fragmental method calculated; Ghose improvement for the Lipinski rule of Five [26], and synthetic accessibility from 1 (very easy) to 10 (very difficult), implemented in the software. The most relevant compounds were selected according to its molecular descriptors and leadlikeness properties. A complete table of all descriptors could be seen in Table S1. Figure 1 depicts the steps for the compound selection.

### 2.2. Docking

Once we chose leader compounds from the previously mentioned list, we used the COVID-19 Docking Server (https://ncov.schanglab.org.cn/), a web server that predicts the binding modes between different COVID-19 targets and the ligands. A complete description of the algorithm used for such could be found [28]. We tested the targets: Main protease, papain-like protease, Nsp3 (AMP site), Nsp3 (MES site), RdRp (RTP site), RdRp (RNA site), Helicase (ADP site), Helicase (NCB site), Nsp14 (ExoN), Nsp14 (N7-MTase), N protein (NCB site) with the selected ligands, accordingly to the best conformation (emodin anthrone, kaempferol, quercetin, aesculin, cichoriin, luteolin, matricin, riolozatrione, monocaffeoyl tartaric acid and aucubin). Complete table of docking results is in Table S2.

### 2.3. Pharmacokinetic Assessment (PBPK Model Building and Evaluation)

Once we chose the best compound according to docking results against SARS-CoV-2, we developed a PBPK model to predict the pharmacokinetic potential of such compounds in an individual. In this sense, the PBPK’s models predict the concentration-time profile of compounds in the body, giving an idea of such compounds’ performance. The adult PBPK model was developed using PK-sim modelling software (version 8.0, 2017, http://www.systems-biology.com/products/pk-sim.html) and according to data from simulation from other coumarins [29] and other variables included in the model could be seen in Table S3.

## 3. Results and Discussion

**Figure 2:**
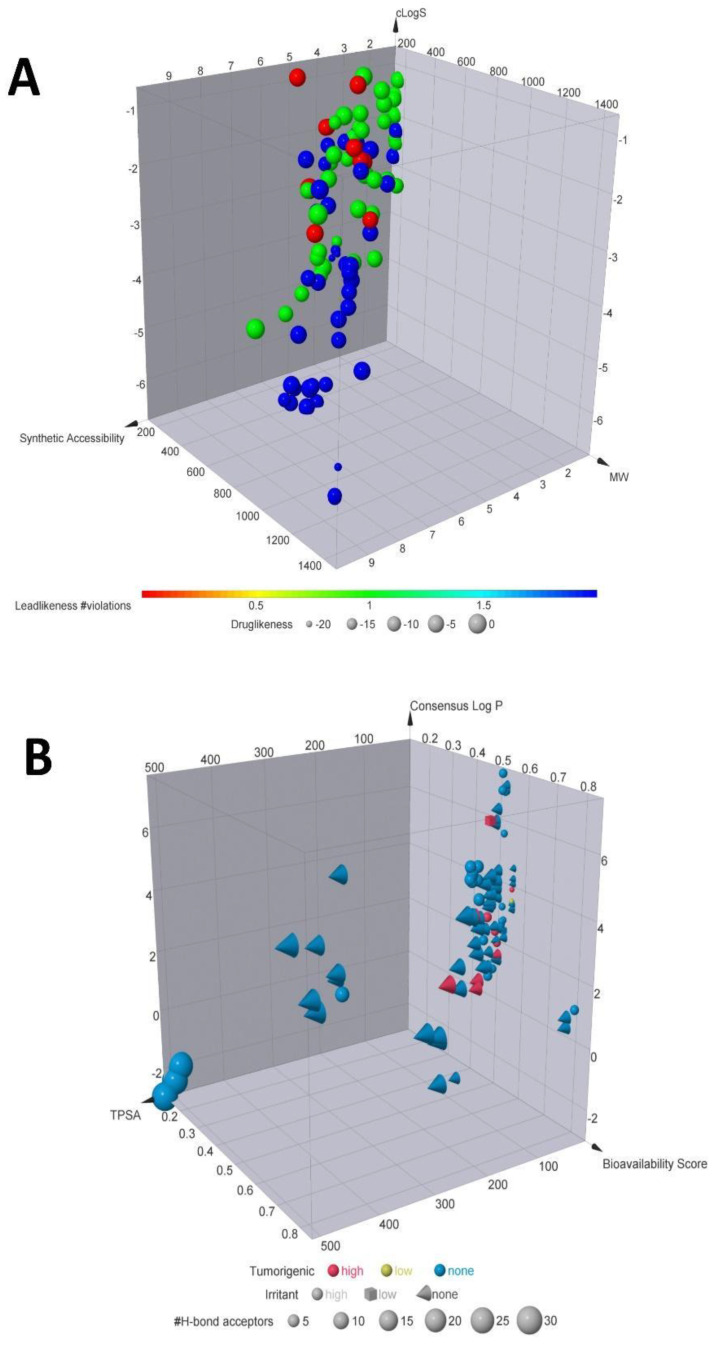
Chemoinformatic properties. (**A**) Molecular descriptors calculated. A selection of some of the most important properties for selected compounds. According to the color code, red dots are compounds with less leadlikeness. In contrast, the blue dots are the compounds with the significant number of leadlikeness, meaning that blue dots may be potential leaders for further analyses. (**B**) Toxicoinformatic properties. Natural products according to their properties such as tumorigenic or irritant effect, Log P, bioavailability score TPSA and H-bonds acceptors.

**Figure 3:**
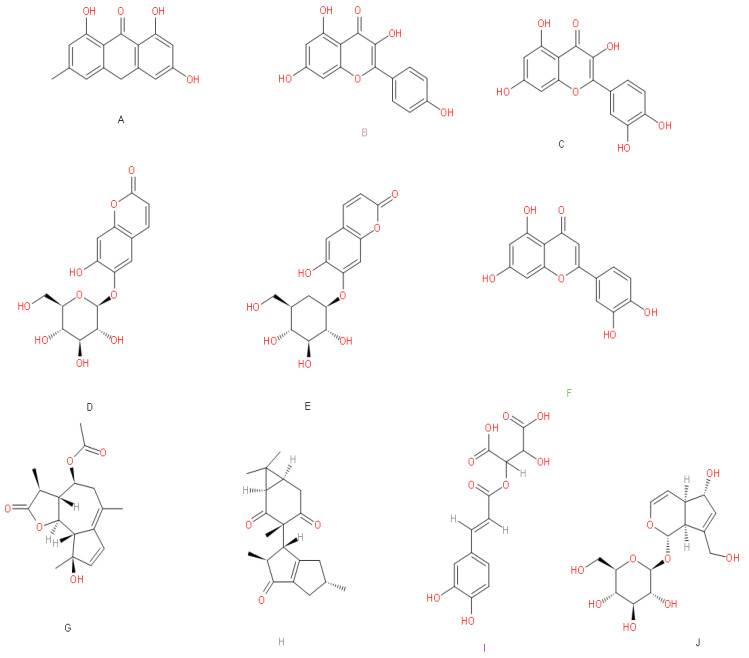
Compounds with chemical properties as leaders. According to the chemoinformatic analysis, we present the ten compounds’ molecular structures that meet the criteria for further analyses, and the Mexican natural materials from which they derive. (**A**) emodin anthrone (*Aloe vera*), (**B**) kaempferol (*Urtica dioica*, *Passiflora incarmata*, *Prunus pérsica* L., *Tilia mexicana* and *Tilia europea*), (**C**) quercetin (*Passiflora incarmata*, *Tilia europea*, *Taraxacum officinale*, *Matricaria recutita*, *Prunus pérsica* L., *Tilia mexicana* and *Urtica dioica*) (**D**) aesculin (*Taraxacum officinale*), (**E**) cichoriin (*Taraxacum officinale*), (**F**) luteolin (*Scoparia dulce* L., *Taraxacum offcinale* and *Passiflora incarmata*), (**G**) matricin (*Matricaria recutita*), (**H**) riolozatrione (*Jatropha dioica*), (**I**) monocaffeoyl tartaric acid (*Taraxacum officinale*), (**J**) aucubin (*Verbascum densiflorum*).

To establish which compounds are the most suitable for drug repurposing against SARS-CoV-2 targets, we calculated the molecular descriptors for each of the 100 compounds (physicochemical features derived from the chemical structures at different dimensions), shown in Figure 2 and Table S1. We also calculated the toxicoinformatic properties, such as LogP, bioavailability score, TPSA, the tumorigenic, mutagenic, reproductive effects and irritant potential (Figure 2B). In this sense, our results indicate that from the 100 compounds, only ten compounds (emodin anthrone, kaempferol, quercetin, aesculin, cichoriin, luteolin, matricin, riolozatrione and monocaffeoyl tartaric acid, Figure 3) meet the leadlikeness and Lipinski’s rules to continue the subsequent analyses against SARS-CoV-2 targets. Interestingly, from these ten selected compounds, several studies suggest them as potential candidates with antibacterial, antifungal or antiviral activity. For instance, a previous study conducted by Azizah et al., demonstrates that luteolin, kaempferol and emodin isolated from the plant *Ventilago deticulata* exhibit antibacterial and antifungal properties against *Staphylococcus aureus*, *Pseudomonas aeruginosa*, *Escherichia coli*, *Bacillus cereus*, *Salmonella enterica*, and fungus such as *Candida albicans* [30].

Regarding, in the antiviral activity, methanolic extracts from both *Taraxacum officinale* and *Urtica dioica* demonstrated to inhibit the replication of dengue virus serotype 2 in vitro [31], authors hypothesized that this effect might be mediated in part by quercetin or luteolin compounds since both compounds have been tested against viruses such as dengue virus, chikungunya virus [32], and coxsackievirus A16 [33], respectively. In this context, kaempferol has been tested against dengue virus and the Japanese encephalitis virus. Interestingly such compound demonstrates to inhibit both viral infections [34], significantly. Moreover, caffeoyl tartaric acids derivatives (monocaffeoyl tartaric acid), coumarins (aesculin and cichoriin) among other compounds found in plants of the genus Taraxacum, including *Taraxacum officinale*, have been described to exhibit promising antibacterial activity against *S. aureus*, *B. cereus* and *E. coli* [35]. Beside antibacterial activity, oligomerization of aesculin modulates antibiotic resistance from E. coli strains resistant to both ofloxacin and amoxicillin, representing a promising strategy for treating antibiotic-resistant strains [36]. Another compound with antiviral activity is riolozatrione, which reduces herpes simplex type 1 and type 2 [24,37]. Finally, monocaffeoyl tartaric acid induces antibacterial and antifungal activities against *S. aureus*, *B. cereus*, *E. coli*, *S. tiphy*, and *C. albicans* [38]. Surprisingly, despite the vast number of references that argue for the matricin’s potential antimicrobial effect, this compound has not been tested alone in vivo or in vitro. The literature only reports the antibacterial effect of extracts derived from Matricaria spp., suggesting for this field an opportunity area for further analyses.

**Figure 4:**
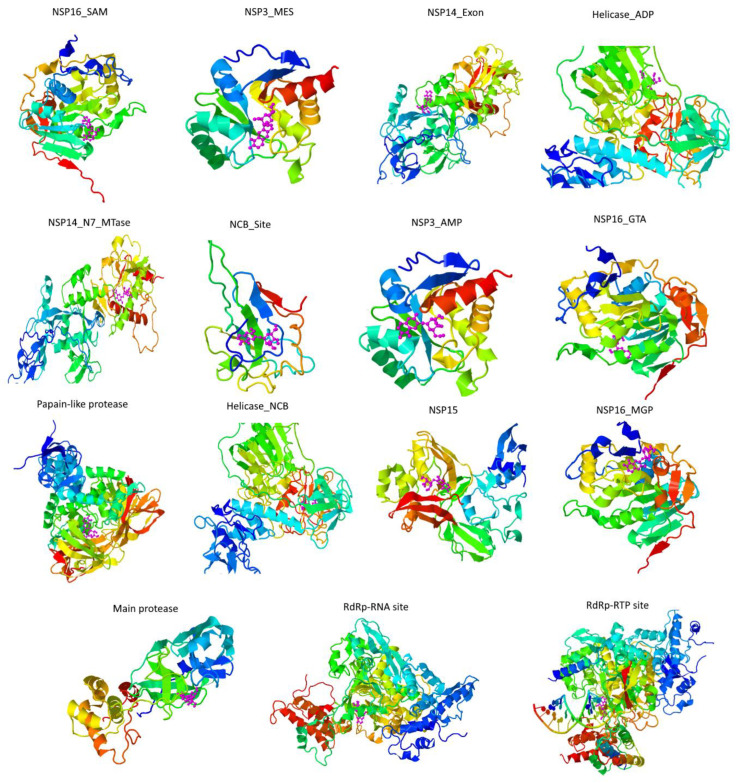
Cichoriin dockings conformations against COVID-19 targets. Cichoriin (magenta) was docked to the main pharmaceutically attractive targets (rainbow ribbon 3D protein structure) of SARS-CoV-2 with the COVID-19 docking server. As depicted in this figure, cichoriin achieves the most stable conformations with against the main protease (papain-like protease), Nsp3 (AMP site), Nsp3 (MES site), RdRp (RTP site), RdRp (RNA site), Helicase (ADP site), Helicase (NCB site), Nsp14 (ExoN), Nsp14 (N7-MTase) and N protein (NCB site).

Once we had identified the compounds that meet the physicochemical criteria to become drug leaders, we evaluated their potential against the different targets of SARS-CoV-2. The results obtained from the COVID-19 Docking Server (Table 1 and Table S2), demonstrate that among the ten leader compounds, just three compounds (quercetin, riolozatrione and cichoriin) achieve the most stable conformation against the main targets of SARS-Cov-2 (the more negative ΔG is, the more the equilibrium of the reaction is on the side of the resulting conformation [39]). However, results from the toxicoinformatic analysis (Figure 2 and Table S1) indicate that riolozatrione could be irritant, mutagenic, tumorigenic, and with possible reproductive effects; quercetin may possess potential as mutagenic and tumorigenic, while such features compromise to continue the subsequent analysis with these compounds. Meanwhile, cichoriin appeared as the safest compound according to its toxicoinformatic properties described in Figure 2 and Table S1. Thus, we only show the conformations achieved by cichoriin with the targets of SARS-CoV-2 in Figure 4. Accordingly, the best targets for cichoriin binding are RdRp (RTP site), Nsp14 (ExoN), Nsp3 (207-379, AMP site) and papain-like protease (Table 1 and Table S2 and Figure 4).

**Figure 5:**
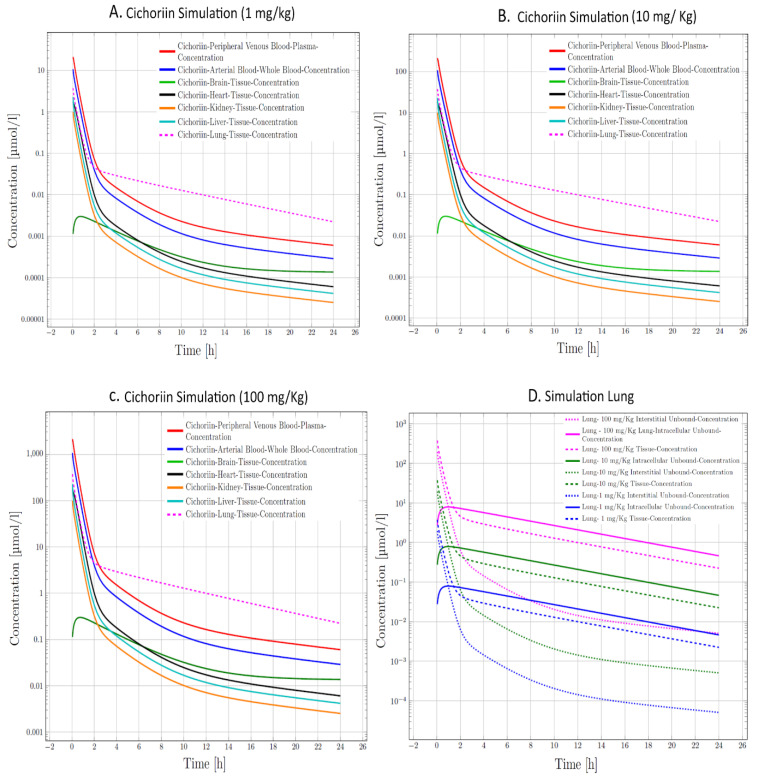
Physiologically based pharmacokinetic model (PBPK) simulation of Cichoriin IV at three concentrations. (**A**) 1 mg/kg, (**B**) 10 mg/kg, (**C**) 100 mg/kg. (**D**) Cichoriin kinetics in the lung with the three selected concentrations, suggesting that the best dose is 100 mg/kg IV (pink).

In our next step in the analysis, we aimed to characterize whether cichoriin had pharmacokinetic potential against COVID-19 since this issue stops several new compounds from going to clinics. However, since cichoriin is not yet in human studies, required data to build the PBPK model simulations was constructed using data from other coumarins with similar structure [29] (complete simulation data could be found in Table S3). We adjust the PBPK model parameters expecting that cichoriin will be used in patients with COVID-19 in critical conditions. Thus, we simulate a 60-year-old male Mexican American with three potential doses of 1, 10, and 100 mg/kg administered IV. The simulation results showed that cichoriin reaches acceptable concentration in arterial, peripheral blood, and intracellularly (Figure 5); the complete data results are in Table S4. Additionally, cichoriin reaches a higher concentration in the lungs intracellular than other compartments, suggesting that the best dose to treat COVID-19 may be 100 mg/Kg. Some of the targets are found once the virus is inside the cell.

Cichoriin is a glycoside member of coumarins and has been isolated in dandelion (*Taraxacum officinale*) and in chicory (*Cichorium intybus*) belonging to the Asteraceae family [40]. Dandelion is distributed ubiquitously in all the geographical regions of Mexico [41], while chicory growth is restricted only to Mexico’s central and northeast region [42]. In this sense, cichoriin may be a promising molecule against COVID-19 not only due to its possible effects against SARS-CoV-2 targets, but also since its anti-inflammatory and antioxidant effects mediated via NF-κB, Akt and the MAP-kinases MEK and ERK [43,44] suggest that this compound may target the chronic proinflammatory cytokine storm harmful for individuals with severe COVID-19 [45]. Additionally, cichoriin has been reported to induce antifungal, antibacterial [46] and photoprotective effects [47,48] and have an average synthetic accessibility score chemical nature as a natural product, suggesting that it may be easy to obtain and may be used for other targets.

## 4. Conclusions

COVID-19 has taken the lives of more than 2 million people worldwide, and to date, there is no effective pharmacological treatment available for such disease (until January 2021). Therefore, in the present work, we performed a chemoinformatic screening with 100 compounds isolated from Mexican natural products to seek active molecules with the potential to be implemented in the pharmacological treatment of such disease (either as a drug itself or as an inspiring molecule to developed active compounds against SARS-CoV-2). In this sense, we found ten compounds isolated from natural products from Mexico with leadlikeness and Lipinski’s potential. However, after the docking and toxicoinformatic analysis, only cichoriin was safe and docked with high affinity to the main targets of SARS-CoV-2. Interestingly, the PBPK simulation showed that this compound might reach acceptable levels in plasma and highest concentration in the lung when administered IV at 100 mg/kg, suggesting that cichoriin may be a potential candidate in treating severe COVID-19.

Nevertheless, despite these promising results, the present study’s main drawback relies on the lack of experimental data and further experimental studies are urgently required to validate our results. However, cichoriin is a glycoside member of coumarins, and in general, coumarins have shown important pharmacokinetic properties that make them easier to implement in clinics. Therefore, we propose that cichoriin be considered for further experimental studies to generate active compounds useful in treating critical cases of COVID-19.

## Figures and Tables

**Table 1:** Docking results for leader compounds against the main targets of SARS-CoV-2. Protease, papain-like protease, Nsp3 (AMP site), NSp3 (MLS site), RdRp (RTP site), RdRp (RNA site), Helicase (ADP site), Helicase (NCB site), Nsp14 (MTase), Nsp14 (Exon), Nsp15 (endoribonuclease), Nsp16 (GTA site), Nsp16 (SAM site), N protein (NCB site). A—aucubin, C—cichoriin, EA—emodin anthrone, E—esculin, K—kaempferol, L—luteoilin, M—matricin, MTA—monocaffeyl tartaric acid, Q—quercetin, R—riolozatione.

	A	C	EA	E	K	L	M	MTA	Q	R
Protease	−6.5	−7.4	−7.1	−7.7	−7.4	−7.3	−7.1	−6.7	−7.3	−6.4
Papain-like protease	−7.7	−8.3	−7.9	−8.7	−8.2	−8.3	−8.5	−7.2	−8.4	−9.4
Nsp3 (207-379, AMP site)	−7.7	−7.1	−7.5	−7.5	−7.8	−8.1	−7.1	−6.8	−8.3	−8.3
Nsp3 (207-379, MES site)	−6.8	−8.4	−8.1	−7.8	−8.6	−7.9	−7.6	−7.7	−8.6	−8.1
RdRp (RTP site)	−9.4	−9.5	−9.2	−9.4	−10.2	−8.4	−7.5	−9	−10.5	−8
RdRp (RNA site)	−6.6	−7.5	-6.7	−7.7	−7.4	−7.5	−7.2	−7	−7.5	−7.5
Helicase (ADP site)	−6.3	−6.7	−6.3	−6.6	−6.3	−6.6	−6.6	−6.3	−6.5	−6
Helicase (NCB site)	−7	−7.4	−7	−7.2	−7.2	−7	−7.1	−7.1	−7.2	−7.7
Nsp14 (ExoN)	−7.8	−8.8	−8.6	−8.3	−8.7	−8.7	−8.1	−7.3	−8.7	−8.9
Nsp14 (N7-MTase)	−6.6	−7.5	−6.8	−7.2	−7.3	−7	−6.1	−6	−7.4	−6.5
Nsp15 (endoribonuclease)	−6.5	−7.2	−7.3	−6.9	−7	−7.2	−6.6	−6.4	−6.8	−7.3
Nsp16 (GTA site)	−7.3	−8.3	−7.9	−7.9	−8.6	−8.8	−7.5	−7	-8.7	−7.5
Nsp16 (MGP site)	−6	−7.2	−7.1	−7.2	−7	−6.7	−6.3	−6.2	−6.9	−7.4
Nsp16 (SAM site)	−7.5	−8.1	−7.5	−8	−8.7	−8.9	−6.8	−7.2	−8.7	−7.4
N protein (NCB site)	−6.6	−7.7	−8.7	−7.8	−8.1	−7.9	−6.5	−6.7	−8.4	−9

## Data Availability

All data are available in the Supplementary Materials.

## Supplementary Materials

The following are available online at https://www.mdpi.com/2218-273X/11/2/216/s1, Table S1: Chemoinformatic results of the 100 selected compounds. Table S2: Complete table of docking simulations. Table S3: Simulation conditions applied for PBPK modelling. Table S4: Complete pharmacokinetic parameters for PBPK modelling.
